# A preliminary exploration of the effect of concurrent antidepressant medication on responses to high-frequency repetitive transcranial magnetic stimulation (rTMS) in severe, enduring anorexia nervosa

**DOI:** 10.1186/s40337-021-00370-3

**Published:** 2021-01-28

**Authors:** Bethan Dalton, Jessica McClelland, Savani Bartholdy, Maria Kekic, Iain C. Campbell, Ulrike Schmidt

**Affiliations:** 1 Section of Eating Disorders, Department of Psychological Medicine, Institute of Psychiatry, Psychology & Neuroscience, King’s College London, London, SE5 8AF UK; 2 South London and Maudsley NHS Foundation Trust, Maudsley Hospital, Denmark Hill, London, SE5 8AZ UK

**Keywords:** Repetitive transcranial magnetic stimulation, Anorexia nervosa, eating disorders

## Abstract

**Background:**

Repetitive transcranial magnetic stimulation (rTMS) is a promising treatment option for people with severe, enduring anorexia nervosa (SE-AN). As depression is often comorbid with AN, antidepressant medication is commonly prescribed to this patient group. Concurrent antidepressant medications may affect the rTMS treatment response. Therefore, in a secondary analysis of data from a feasibility trial, we explored the effect of antidepressant medication on responses to rTMS in people with SE-AN.

**Methods:**

Twenty-six participants with SE-AN received 20 sessions (over 4 weeks) of neuronavigated high-frequency real rTMS to the left dorsolateral prefrontal cortex. Eating disorder (ED) and general psychopathology symptoms were assessed at baseline, post-treatment and at a 4-month follow-up.

**Results:**

Participants taking antidepressants (*n* = 16) for the duration of the rTMS treatment had a greater reduction in ED symptoms at the 4-month follow-up, compared to those not taking antidepressants (*n* = 10). Antidepressant status was not however associated with significant changes in mood outcomes.

**Conclusions:**

In people with SE-AN receiving rTMS treatment, preliminary findings suggest a potential synergistic effect of antidepressant medication in reducing ED symptomatology. There was however no associated reduction in affective symptoms i.e., the effect does not seem to be related to changes in mood. The sample in this exploratory study was small and heterogenous. However, the preliminary results provide a basis for hypothesis generation for future studies.

**Supplementary Information:**

The online version contains supplementary material available at 10.1186/s40337-021-00370-3.

## Plain English summary

Approximately 20–30% of patients with anorexia nervosa have a severe and enduring form of the illness. Therefore, new treatment approaches are needed. Repetitive transcranial magnetic stimulation (rTMS) is a promising treatment option for people with severe, enduring anorexia nervosa. Research in people with depression suggests that taking antidepressants may affect a person’s response to rTMS treatment. People with anorexia nervosa often experience depression and antidepressants are commonly prescribed to this patient group. This study explored the effect of antidepressants on responses to rTMS in people with severe, enduring anorexia nervosa. Participants received 20 sessions (over 4 weeks) of rTMS. We found that those taking antidepressants during rTMS treatment showed a greater reduction in eating disorder symptoms at follow-up, compared to those not taking antidepressants. Participants not taking antidepressants and those taking antidepressants showed similar improvements in depression and anxiety symptoms. This suggests that the improvement in eating disorder symptoms associated with the combination of rTMS and antidepressants was not related to changes in mood. The study sample was small, and we were unable to explore the effect of other medications on response to rTMS treatment. Further research is needed to investigate the effect of antidepressant medication on response to rTMS treatment in this patient group.

## Background

Repetitive transcranial magnetic stimulation (rTMS) is a promising treatment option for severe, enduring anorexia nervosa (SE-AN; [1]). In a recent feasibility clinical trial (the TIARA study; [2]), we found small effects of rTMS on body mass index (BMI) and eating disorder (ED) symptoms, together with moderate to large positive effects on mood outcomes at follow-up (Cohen's *d* = 0.61 to 1.0; 4-months post-randomisation), favouring real rTMS over sham, in people with SE-AN [3]. It has been proposed that concurrent antidepressant medication use may influence rTMS treatment outcomes in major depressive disorder (MDD; [4]). A higher proportion of participants (61.7%) in the original TIARA trial were taking antidepressant medication, compared to those in other treatment trials in AN (e.g., [5, 6]). Therefore, using data from the TIARA trial (and the subsequent open trial), we explored whether rTMS treatment response was affected by concurrent antidepressant medication.

## Methods

Full methods of the TIARA trial are described elsewhere ([2, 3]; trial registration number: ISRCTN14329415, pre-results). The study was a double-blind, randomised sham-controlled feasibility trial in adult women with SE-AN. Participants were randomly allocated to 20 sessions of real or sham neuro-navigated high-frequency rTMS delivered to the left dorsolateral prefrontal cortex (DLPFC). Participants in the real group received rTMS at the following parameters: 110% resting motor threshold; 10 Hz frequency consisting of twenty 5-s trains with 55-s inter-train intervals (1000 pulses/session) with a total duration of 20 min (see Supplementary S1 for further details). Sham stimulation was administered at the same parameters as real rTMS; however, a sham coil was used. Ethical approval was given by the London – City Road & Hampstead Research Ethics Committee (Reference: 15/LO/0196).

Clinical outcomes were assessed in all participants at baseline (pre-randomisation), within 1 week of completing rTMS treatment (post-treatment; 1-month post-randomisation) and at a follow-up (4-months post-randomisation). Outcomes used in the present analyses were related to ED symptoms, assessed by BMI and the Eating Disorder Examination – Questionnaire (EDE-Q; [7]), and mood, as measured by the Depression Anxiety Stress Scales – Version 21 (DASS-21; [8]). Medication status was recorded at screening and confirmed at start of rTMS treatment.

Participants were unblinded following completion of data collection at the follow-up assessment. If they remained eligible, those who completed sham treatment were given the opportunity to receive real rTMS treatment (using the identical protocol and rTMS parameters to that used in the main trial, described above). Participants who took this opportunity were re-screened and completed baseline, post-treatment, and follow-up assessments at the same schedule as described above as part of an open study. For the present study, only participants who received real rTMS, either in the initial trial (originally allocated to real rTMS) or the associated open study (originally allocated to sham rTMS but completed real rTMS), were included.

Data analyses were performed in Stata 15 [9]. Depending on the distribution of the data, between-subjects t-tests or Mann-Whitney U tests were used to assess group differences in baseline demographic and clinical characteristics. A chi-squared test was used to examine group differences in AN-type. The post-treatment and follow-up group means and SDs for the clinical outcomes were adjusted for baseline (post-treatment/follow-up scores minus baseline scores) and have been presented with between-group effect sizes (Cohen’s *d*) alongside 95% confidence intervals.

## Results

Twenty-six participants received real rTMS and were included in this study (*n* = 16 initially randomised to real rTMS treatment, *n* = 10 completed real rTMS treatment as part of the open study after initially being randomised to sham rTMS). Sixteen participants were taking a stable dose of antidepressant medication for the duration of rTMS treatment (data from one participant is missing at follow-up) and ten were not taking antidepressants. In the antidepressant group, ten were taking only a selective serotonin reuptake inhibitor (*n* = 1 Fluvoxamine, *n* = 2 Fluoxetine, *n* = 7 Sertraline), two were taking both a norepinephrine–dopamine reuptake inhibitor and a selective serotonin reuptake inhibitor (*n* = 1 Bupropion and Sertraline, *n* = 1 Bupropion and Fluoxetine), two were taking a selective serotonin and norepinephrine reuptake inhibitor (Duloxetine), one was taking a monoamine oxidase inhibitor (Phenelzine), and one was taking a tetracyclic antidepressant (Mirtazapine).

Full medication details and other concurrent treatments are detailed in Supplementary S2. Baseline demographics for both groups are shown in Table 1. Groups did not significantly differ in demographic or clinical characteristics at baseline (*p* > 0.05), although the antidepressant group reported more severe ED, depression and anxiety symptoms. Clinical characteristics at post-treatment and follow-up are presented in Supplementary S3.

**Table 1:** Baseline demographics and clinical characteristics for the antidepressant and no antidepressants groups

	Antidepressants (***n*** = 16)	No antidepressants (***n*** = 10)	Group comparison
Age (years) (mean ± SD)	29.56 ± 9.15	33.20 ± 13.29	*U* = 64.5, z = 0.82, *p* = 0.413
Illness duration (years) (mean ± SD)	14.16 ± 9.38	19.40 ± 14.13	*U* = 61.5, z = 0.98, *p* = 0.328
AN-R / AN-BP (*n*)	10 / 6	6 / 4	χ^2^(1) = 0.02, *p* = 0.899
BMI (kg/m^2^) (mean ± SD)	16.36 ± 1.50	15.95 ± 1.85	*t*(24) = −0.62, *p* = 0.540
EDE-Q Global (mean ± SD)	4.31 ± 0.82	3.64 ± 1.58	*t*(24) = − 1.45, *p* = 0.164
EDE-Q Restraint (mean ± SD)	4.20 ± 1.34	3.42 ± 1.86	*t*(24) = − 1.25, *p* = 0.225
EDE-Q Eating Concern (mean ± SD)	3.69 ± 0.80	3.28 ± 1.88	*t*(24) = − 0.77, *p* = 0.449
EDE-Q Shape Concern (mean ± SD)	4.83 ± 1.02	4.08 ± 1.68	*U* = 59, z = − 1.11, *p* = 0.266
EDE-Q Weight Concern (mean ± SD)	4.53 ± 0.98	3.78 ± 1.64	*t*(24) = − 1.46, *p* = 0.157
DASS-21 Total (mean ± SD)	74.00 ± 24.61	62.60 ± 29.33	*t*(24) = − 1.07, *p* = 0.296
DASS-21 Depression (mean ± SD)	29.13 ± 9.72	22.00 ± 10.71	*t*(24) = − 1.75, *p* = 0.093
DASS-21 Anxiety (mean ± SD)	17.38 ± 10.22	12.60 ± 10.96	*t*(24) = − 1.13, *p* = 0.865

*Abbreviations*: *SD* Standard deviation, *AN-R* Anorexia nervosa restricting type, *AN-BP* Anorexia nervosa binge-eating/purging type, *n* Number, *BMI* Body mass index, *EDE-Q* Eating Disorder Examination – Questionnaire, *DASS-21* Depression, Anxiety and Stress Scales – Version 21

The means, standard deviations and between-group treatment effect sizes (with confidence intervals) for change scores (baseline to post-treatment and baseline to follow-up) of the clinical outcomes are shown in Table 2. Post-treatment group differences in clinical outcomes were of small effect: ED symptom outcomes favoured rTMS with concurrent antidepressant medication whereas BMI and mood outcomes favoured rTMS alone (i.e., no concurrent antidepressant medication). At follow-up however, there were large between-group effect sizes in measures of ED symptoms and small to medium effect sizes for BMI and mood outcomes. These all favoured rTMS treatment with concurrent antidepressant medication.

**Table 2:** The mean change scores (post-treatment and follow-up scores adjusted for baseline^a^) for the clinical outcome measures, including the number of participants (N), means, and standard deviations (SD) for the antidepressant and no antidepressant groups, and the estimated between-groups effect size (Cohen’s *d* with 95% confidence intervals)

Measure	Antidepressants			No antidepressants			Between- subject ***d*** (95% CI)
	*N*	Mean	SD	*N*	Mean	SD	
**Post-treatment (adjusted for baseline**^a^**)**							
BMI	16	0.03	0.72	10	0.16	0.36	0.22 (− 0.58 to 1.01)
EDE-Q Global	16	− 0.34	0.68	10	− 0.09	0.56	0.40 (−0.41 to 1.19)
EDE-Q Restraint	16	−0.18	0.73	10	0.14	0.67	0.45 (−0.36 to 1.24)
EDE-Q Eating Concern	16	−0.43	0.83	10	−0.12	0.84	0.37 (−0.43 to 1.16)
EDE-Q Shape Concern	16	−0.45	0.74	10	−0.10	1.11	0.38 (−0.42 to 1.18)
EDE-Q Weight Concern	16	−0.33	0.83	10	−0.30	0.94	0.03 (−0.76 to 0.82)
DASS-21 Total	16	−9.00	22.14	10	−16.2	19.31	−0.34 (−1.13 to 0.46)
DASS-21 Depression	16	−1.25	8.61	10	−4.80	8.34	−0.42 (−1.21 to 0.39)
DASS-21 Anxiety	16	−3.63	8.86	10	−4.20	4.76	−0.08 (− 0.87 to 0.72)
**Follow-up (adjusted for baseline**^a^**)**							
BMI	15	0.27	1.17	10	0.06	0.89	−0.20 (−1.00 to 0.91)
EDE-Q Global	15	−0.88	0.85	10	−0.03	0.62	**1.10 (0.23 to 1.96)**
EDE-Q Restraint	15	−0.75	1.29	10	0.18	0.68	**0.85 (0.001 to 1.67)**
EDE-Q Eating Concern	15	−0.67	1.06	10	0.06	0.95	0.71 (−0.12 to 1.53)
EDE-Q Shape Concern	15	−1.06	0.83	10	−0.09	0.83	**1.17 (0.29 to 2.03)**
EDE-Q Weight Concern	15	−1.04	1.01	10	−0.28	0.67	**0.85 (0.006 to 1.68)**
DASS-21 Total	15	−17.33	23.80	10	−14.4	18.40	0.13 (−0.69 to 0.93)
DASS-21 Depression	15	−8.13	10.43	10	−4.20	8.24	0.41 (−0.41 to 1.21)
DASS-21 Anxiety	15	−4.40	7.57	10	−3.00	7.13	0.19 (−0.62 to 0.99)

*Abbreviations*: *N* Number, *SD* Standard deviation, *CI* Confidence intervals, *BMI* Body mass index, *EDE-Q* Eating Disorder Examination – Questionnaire, *DASS-21* Depression Anxiety and Stress Scales – Version 21

^a^Post-treatment/follow-up scores minus baseline scores. Bold font signifies that the CI do not include 0

We further explored the association between antidepressant use and changes in ED symptoms by calculating ED symptom recovery rates (EDE-Q Global Score < 2.77, i.e., < 1 SD above community mean; [10]) for each group. In the antidepressant group at baseline, no participants were classed as recovered in terms of ED symptoms. ED symptom recovery was reported in 3/16 (18.75%) participants at post-treatment and 6/15 (40%) participants (including the *n* = 3 recovered at post-treatment) at follow-up. In the no antidepressant group, of those who did not report ED symptom recovery prior to rTMS treatment (*n* = 7, 70%), one additional participant reported ED symptom recovery at post-treatment only (1/7, 14.29%) and one further participant at follow-up only (1/7, 14.29%).

## Discussion

This small exploratory study examined the potential effects of antidepressant medication on responsiveness to rTMS treatment in people with SE-AN. These preliminary findings show that concurrent antidepressant medication is not associated with ED outcomes immediately post-rTMS treatment nor with mood-related outcomes at post-treatment or follow-up. In fact, at follow-up, in the antidepressant group, depression scores remained in the severe range (according to clinical cut-off scores on the DASS-21; [8]). However, in relation to ED symptoms, there were large between-group effect sizes on change scores from pre-treatment to follow-up: these favoured the group with real rTMS and concurrent antidepressant medication. Furthermore, following rTMS treatment, a greater number of participants in the antidepressant group reported ED symptom recovery compared to those who were not on concurrent antidepressant medication. This is of particular relevance given the somewhat limited treatment success usually seen in SE-AN.

Antidepressants are commonly prescribed to adults with AN [11]. There is some evidence that they improve symptoms of comorbid depression in AN [12], but limited data for them being effective in improving ED symptoms [13]. In the TIARA trial, ~ 60% of our participants were taking antidepressants and in fact, the total group had somewhat higher depression scores compared to other treatment studies of AN (e.g., [5]). In the present analyses, those taking antidepressants reported more severe ED, depression and anxiety symptoms at baseline i.e., they appeared to be more psychologically unwell (though none of the differences were statistically significant). In this context, it is of note that none of the participants in the antidepressant group, but 3/10 (30%) of the participants in the no antidepressant group, reported ED symptom recovery prior to rTMS treatment.

In MDD, rTMS is typically administered as an augmentation therapy to antidepressant medication (e.g., [14]). However, little is known about the potential impact of antidepressants on the clinical efficacy of rTMS (and vice versa). Data on the effects of concurrent antidepressant medication during rTMS treatment for MDD is mixed, reporting either an enhancement of the rTMS response [4, 15] or no effect [16]. In terms of potential synergistic mechanisms, it is of note that both rTMS and antidepressant medication (including those with rapid onset effects) have been reported to alter neuronal plasticity and cortical excitability [17–20]. To an extent, this is suggested by our data. It is unclear however, why antidepressant status was associated with improvements in ED outcomes, but not with significant changes in mood following the rTMS treatment, especially as symptoms of depression are the typical target of antidepressants and these medications are reported to have little efficacy in treating ED symptoms [13].

Our study has some limitations: our sample size was small and it is an exploratory analysis of pre-existing data, i.e., concurrent antidepressant medication and dose were not standardised across participants and a proportion in both groups were also taking other psychotropic medications. It was not possible to explore whether other concurrent medications (e.g., benzodiazepines) were associated with rTMS response in SE-AN due to the small number of participants taking these. Recent research suggests that in response to rTMS treatment in MDD, benzodiazepine use is associated with less improvement and psychostimulants with greater improvement [16, 21]. For these exploratory analyses, we combined participants who received real rTMS from the original randomised controlled trial (i.e., participants were blinded) with those who received real rTMS as part of the open label follow-up (i.e., participants were unblinded). However, there was approximately equal distribution of these across the no antidepressant and antidepressant groups (e.g., 60–62.5% in both groups received real rTMS in the original randomised controlled trial). In addition, we identified a possible floor effect in the no antidepressant group in terms of ED symptom recovery, where a limited potential for further symptom reductions in these participants may have contributed to the findings. Furthermore, we did not systematically assess comorbid psychiatric diagnoses (e.g., depression). Given the relatively short follow-up period, longer term follow-ups are needed to examine whether the observed effects persist and/or extend to other clinical features. There is likely also value in exploring the combination of rTMS with other treatment approaches, aside from medication in SE-AN, such as psychotherapy or cognitive training [22].

## Conclusions

In people with SE-AN, who received 20 sessions of high-frequency rTMS to the left DLPFC, we have obtained preliminary findings that at follow-up, antidepressant medication appears to enhance the response to rTMS with respect to ED symptoms but not to affective symptoms. The impact of concurrent medication on rTMS efficacy needs to be more rigorously tested in larger samples of people with SE-AN and these preliminary results provide the basis for hypothesis generation for these future studies.

## Supplementary Information

**Additional file 1.**

## Data Availability

The datasets generated and/or analysed during the current study are not publicly available but are available from the corresponding author on reasonable request.

## Abbreviations

AN: Anorexia nervosa
BMI: Body mass index
DASS-21: Depression Anxiety and Stress Scales – Version 21
ED: Eating disorder
EDE-Q: Eating Disorder Examination Questionnaire
MDD: Major depressive disorder
rTMS: Repetitive transcranial magnetic stimulation
SE-AN: Severe enduring anorexia nervosa
