# Supplementary material for: A preliminary exploration of the effect of concurrent antidepressant medication on responses to high-frequency repetitive transcranial magnetic stimulation (rTMS) in severe, enduring anorexia nervosa
Source: J Eat Disord. 2021 Jan 28;9:16. doi: 10.1186/s40337-021-00370-3 (PMC7845115; doi:10.1186/s40337-021-00370-3)
Supplement: Supplementary file 1 — Additional file 1. [file 40337_2021_370_MOESM1_ESM.pdf]

A preliminary exploration of the effect of concurrent antidepressant medication on responses to high-frequency repetitive transcranial magnetic stimulation (rTMS) in severe, enduring anorexia nervosa

Bethan Dalton, Jessica McClelland, Savani Bartholdy, Maria Kekic, Iain C. Campbell & Ulrike Schmidt

Supplementary file

S1: rTMS target and parameters

Target: The left dorsolateral prefrontal cortex (DLPFC) was selected as the target brain area in this trial for several reasons: (a) the DLPFC is involved in a number of cognitive processes that have been implicated in anorexia nervosa (AN) e.g., emotion regulation, self-control (described in: [1, 2]); (b) high-frequency rTMS to the left DLPFC has shown efficacy and acceptability in the treatment of related psychiatric disorders, including depression [3]; and (c) for practical accessibility reasons.

In order to localise the DLPFC (Talairach co-ordinates x = −45 y =45 z = 30) [4, 5] for neuronavigation (using Brainsight™ neuronavigation software) purposes, all participants underwent a structural MRI scan prior to starting rTMS treatment.

Parameters: To determine the intensity of the rTMS stimulation, a Magstim Rapid device (Magstim®, Whitland, Wales, UK) with a real TMS figure-of-eight coil was used to determine participants' motor threshold (MT). This represents membrane-related excitability of cortical axons. Using the motor-evoked potential method, the MT was established by determining the minimum stimulator output intensity required to obtain five out of ten motor-evoked potentials >50 µV. MT was acquired weekly for each participant to ensure accuracy of the rTMS dose.

The Magstim Rapid device and Magstim D70-mm air-cooled real and sham coils were used to administer real and sham rTMS. Participants in the real group received 20 sessions of high-frequency (10 Hz) rTMS at 110 % of their individual MT, consisting of twenty 5-second trains with 55-second inter-train intervals delivered to the left DLPFC (a total of 1000 pulses delivered over each 20 minute session) [4, 5]. Sham stimulation was administered at the same parameters as real rTMS using a sham coil. The sham coil produces the same noises and feelings as the real coil but does not deliver active stimulation to the brain; rather it stimulates facial and scalp nerves.

S2: Medication and concurrent treatment

Table 1. Psychotropic medication and psychological treatment received concurrently to real rTMS treatment. Each row represents an individual participant. Psychotropic medications remained at a stable dose throughout rTMS treatment.

Antidepressant medication	Other psychotropic medication	Concurrent ED treatment
**No antidepressant group**		
-	Diazepam<br>Nitrazepam	-
-	-	-
-	-	Outpatient
-	-	Outpatient
-	-	Outpatient
-	-	Outpatient
-	-	-
-	-	Outpatient
-	-	Outpatient
-	-	Outpatient
**Antidepressant group**		
Sertraline<br>Bupropion	-	Outpatient
Sertraline	-	Day patient
Fluoxetine	-	Outpatient
Sertraline	-	Outpatient
Fluvoxamine	Aripiprazole<br>Diazepam	-
Sertraline	-	Outpatient
Sertraline	-	-
Phenelzine	-	Outpatient
Fluoxetine	-	Outpatient
Mirtazapine	Pregabalin	-
Duloxetine	Diazepam	-
Duloxetine	Quetiapine	Outpatient
Sertraline	-	Outpatient
Bupropion<br>Fluoxetine	Zolpidem	-
Sertraline	-	Outpatient
Sertraline	-	-

Abbreviations: ED = eating disorder.

S3. Clinical characteristics at post-treatment and follow-up

Table 2. Means and standard deviations of clinical outcome measures at post-treatment and follow-up for the antidepressant and no antidepressant groups.

	Antidepressants (n=16)		No antidepressants (n=10)	
**Measure**	**Post-treatment**	**Follow-up**	**Post-treatment**	**Follow-up**
BMI (kg/m^2) (mean ± SD)	16.39 ± 1.84	16.53 ± 2.24*	16.11 ± 1.78	16.01 ± 2.28
EDE-Q Global (mean ± SD)	3.97 ± 1.14	3.52 ± 1.24*	3.54 ± 1.52	3.61 ± 1.65
EDE-Q Restraint (mean ± SD)	4.03 ± 1.61	3.63 ± 1.46*	3.56 ± 1.72	3.60 ± 1.72
EDE-Q Eating Concern (mean ± SD)	3.26 ± 1.09	3.11 ± 1.25*	3.16 ± 1.58	3.34 ± 1.91
EDE-Q Shape Concern (mean ± SD)	4.38 ± 1.26	3.81 ± 1.57*	3.98 ± 1.45	3.99 ± 1.57
EDE-Q Weight Concern (mean ± SD)	4.20 ± 1.35	3.55 ± 1.48*	3.48 ± 1.66	3.50 ± 1.74
DASS-21 Total (mean ± SD)	65.00 ± 26.56	57.20 ± 31.35*	46.4 ± 36.07	48.20 ± 35.70
DASS-21 Depression (mean ± SD)	27.88 ± 9.67	21.73 ± 12.98*	17.20 ± 13.24	17.80 ± 12.45
DASS-21 Anxiety (mean ± SD)	13.75 ± 13.89	12.40 ± 11.29*	8.40 ± 11.19	9.60 ± 11.99

*n=1 missing. Abbreviations: SD, standard deviation; BMI, body mass index; EDE-Q, Eating Disorder Examination – Questionnaire; DASS-21, Depression Anxiety and Stress Scales – Version 21

References in Supplementary File

1. Dunlop KA, Woodside B, Downar J. Targeting neural endophenotypes of eating disorders with non-invasive brain stimulation. Frontiers in Neuroscience. 2016;10:30. doi: 10.3389/fnins.2016.00030

2. McClelland J, Kekic M, Bozhilova N, Nestler S, Dew T, Van den Eynde F, et al. A randomised controlled trial of neuronavigated repetitive transcranial magnetic stimulation (rTMS) in anorexia nervosa. PloS One. 2016;11:e0148606. doi: 10.1371/journal.pone.0148606

3. Brunoni AR, Chaimani A, Moffa AH, Razza LB, Gattaz WF, Daskalakis ZJ, et al. Repetitive transcranial magnetic stimulation for the acute treatment of major depressive episodes: a systematic review with network meta-analysis. JAMA Psychiatry. 2017;74:143-52. doi: 10.1001/jamapsychiatry.2016.3644

4. Fitzgerald PB, Hoy K, McQueen S, Maller JJ, Herring S, Segrave R, et al. A randomized trial of rTMS targeted with MRI based neuro-navigation in treatment-resistant depression. Neuropsychopharmacology. 2009;34:1255-62. doi: 10.1038/npp.2008.233

5. McClelland J, Kekic M, Campbell IC, Schmidt U. Repetitive transcranial magnetic stimulation (rTMS) treatment in enduring anorexia nervosa: a case series. European Eating Disorders Review. 2016;24:157-63. doi: 10.1002/erv.2414
